# First mitochondrial genomes in the *Effusa* clade (Gastropoda, Ampullariidae): unveiling new insights from Neotropical endemic *Pomacea* spp.

**DOI:** 10.1186/s12864-026-13191-y

**Published:** 2026-07-23

**Authors:** Alejandro Mendivil, Rina Ramírez, Jaime Morin, Jorge L. Ramirez, Betty Shiga, Nilda Oliveros, Carlos Congrains, Michael D. Martin

**Affiliations:** 1 Facultad de Ciencias Biológicas, Universidad Nacional Mayor de San Marcos, Av. Carlos Germán Amezaga 375, Lima, 15081 Peru; 2 Museo de Historia Natural, Universidad Nacional Mayor de San Marcos, Av. Arenales 1256, Lima, 15046 Peru; 3 Department of Natural History, NTNU University Museum, Norwegian University of Science and Technology (NTNU), Erlings Skakkes gate 47B, Trondheim, 7012 Norway; 4 Department of Plant and Environmental Protection Services, University of Hawaii at Manoa, Honolulu, HI 96822 USA

**Keywords:** Apple snails, Mitochondria, Secondary structure, Phylogeny

## Abstract

**Background:**

*Pomacea* is the most diverse genus of the freshwater snail family Ampullariidae, which have been proposed as model organisms in biogeography, speciation and invasive biology. While substantial effort has been put into the mitochondrial genomes of *Pomacea* clades associated with invasive species (*P. canaliculata* and *P. bridgesii* groups), comparatively little is known about the mitogenomic organization and structure within the *Effusa* clade.

**Results:**

This study provides the first complete and annotated mitochondrial genomes of the *Effusa* clade, based on *Pomacea nobilis* (Reeve, 1856) and a putative undescribed *Pomacea* lineage (hereafter *Pomacea* sp.). The mitochondrial genome of *P. nobilis* (16,279 − 16,471 bp) and *Pomacea* sp. (16,355 bp) are among the largest recorded for *Pomacea* and contain the typical 37 animal genes. A comparative analysis with other Ampullariidae revealed that the *Effusa* clade differs from both *P. canaliculata* and *P. bridgesii* clades mainly by the higher proportion of intergenic regions, the size of many genes (*ATP8*, *ATP6*, *NAD6*, *COIII* and *NAD2*), the largest ribosomal RNAs among *Pomacea* and a putative large repetitive unit in the control region.

**Conclusion:**

These findings highlight the distinctiveness of *Effusa* clade mitogenomes in comparison with the *P. canaliculata* and *P. bridgesii* groups. Further mitogenomic studies on underexplored *Pomacea* lineages will be important to broaden our understanding of mitochondrial genome diversity among apple snails.

**Supplementary Information:**

The online version contains supplementary material available at https://doi.org/10.1186/s12864-026-13191-y.

## Background

Ampullariidae (Mollusca, Gastropoda, Caenogastropoda) is a primarily circumtropical family of freshwater snails that reach their highest diversity in the New World, with *Pomacea* Perry, 1810 as the most diverse genus [1, 2]. Due to their Gondwanan origin, large number of species and wide range of ecological and physiological adaptations, apple snails have been proposed as molluscan models for understanding biogeography, speciation and evolutionary adaptations [1]. Additionally, some *Pomacea* species have been introduced and established outside their native range [2–3], and recent population genetic studies have examined their genetic diversity and structure in Southeast Asia and China [4, 5], where invasive apple snails are significant agricultural pests. While the taxonomic identification of *Pomacea* species has traditionally relied on shell characters [6], molecular studies, particularly those based on mitochondrial data, have proven useful to clarify the systematics of very similar species [7, 8]. For example, the mitochondrial gene *COI* was instrumental in distinguishing *Pomacea maculata* Perry, 1810 from *Pomacea canaliculata* (Lamarck, 1822), facilitating the identification of species-diagnostic morphological characters [8].

The mitochondrial genome has been widely used as a genetic marker for phylogenetic and evolutionary studies [9–12] due to its relatively small size compared to the nuclear genome, conserved genetic content and high number of copies per cell [13]. Although the typical animal mitogenome size ranges from 16 to 20 kbp and contains 37 genes organized in 13 protein-coding genes (PCGs), 2 ribosomal RNAs (rRNAs) and 22 transfer RNAs (tRNAs) [14], substantial variation in mitochondrial genome size has been observed among Mollusca [15], including some of the largest mitogenomes recorded in Metazoa (*Anadara kagoshimensis*, 56170 bp [16]). Additionally, gene order rearrangements have been noted in many groups [17, 18], showing potential for elucidating the evolutionary history of freshwater mussels, particularly in Unionoidea [19].

Based on molecular data, *Pomacea* has been classified into four major clades: the *P. canaliculata*,* P. bridgesii*, *Effusa* and *Flagellata* groups [20]. Currently, complete mitogenomes are available on GenBank only for the *P. canaliculata* and *P. bridgesii* groups (search conducted on December 12, 2025). These efforts mainly reflect the relevance of *Pomacea canaliculata* (Lamarck, 1822) and *Pomacea maculata* Perry, 1810 as invasive species, and *Pomacea diffusa* Blume, 1957 as aquatic pet [3], although initiatives focusing on non-introduced species of *Pomacea* have recently increased [21]. In contrast, the *Effusa* and *Flagellata* clades remain underrepresented in mitochondrial data. Hayes et al. [20]. included only two species within the *Effusa* group, *Pomacea glauca* (Linnaeus, 1758) and *Pomacea guyanensis* (Lamarck, 1822), but more recently *Pomacea nobilis* (Reeve, 1856) has also been confirmed as part of this clade [22]. This species is native to South America, and its meat is highly valued and widely consumed in the Peruvian Amazon [23]. The objective of this research was to sequence, assemble, and annotate the mitochondrial genomes of *P. nobilis* and a putative undescribed *Pomacea* lineage from the Aguaytía River (hereafter *Pomacea* sp.), representing the first mitogenomes within the *Effusa* clade. We then incorporated these new data into the *Pomacea* dataset to compare the structure and organization of apple snail mitogenomes (including *P. canaliculata* and *P. bridgesii* clades). This study provides valuable insights into Ampullariidae systematics and *Pomacea* mitochondrial genome evolution.

## Materials and methods

### Specimen collection and DNA extraction

Live specimens of *P. nobilis* and *Pomacea* sp. were collected in the Peruvian Amazon (Table 1) and maintained in tap water, with lettuce provided *ad libitum*, in the Molecular Systematics and Phylogeography Laboratory at Universidad Nacional Mayor de San Marcos. Specimens were relaxed with ethanol, euthanized through thermal shock (4 °C) and immediately preserved in 96% ethanol at room temperature. Genomic DNA was extracted from muscle tissue using the E.Z.N.A. Mollusc DNA Kit (OMEGA Bio-tek) according to the manufacturer’s instructions. Isolated genomic DNA was quantified with the Nanodrop Lite Spectrophotometer (ThermoFisher Scientific). We obtained COI sequences from three *P. nobilis* (GenBank acc. num. MN325113, PX672272, PX672273) and two *Pomacea* sp. specimens (GenBank acc. num. PX672274 and PX672275), following the protocols of Ramírez et al. [22, 23]. These sequences were compared against the NCBI Core nucleotide database (core_nt) using BLASTn [24] with default parameters to assess their taxonomic affinities and exclude possible contamination or misidentifications. To avoid redundancy, only the highest-identity BLASTn hit for each *Pomacea* species was included in Additional file 1. All five specimens were processed for whole genome sequencing and deposited as vouchers in the Museo de Historia Natural of the Universidad Nacional Mayor de San Marcos, Lima, Peru (Table 1).

**Table 1:** Sampling information and mitochondrial genome characteristics

Taxon	Location	Specimen voucher	GenBank accession	Mitogenome size (bp)
*Pomacea nobilis* (Reeve, 1856)	Shebonal, Huallaga River, Loreto, Peru (5°12’18"S, 75°39’22"W)	AE173SHE	PX692142	16,459
	Quebrada Pahuaquiro, Marañón River, Loreto, Peru	AE174PAH	PX692143	16,471
	Cocha Mullaca, Marañón River, Loreto, Peru (4°35’10.93"S, 73°36 × 32.24”W)	Z58Mul	PX692144	16,279
*Pomacea* sp.	Aguaytía, Aguaytía River, Ucayali, Peru (9˚6’55.9"S, 75˚30’41.4"W)	X60AGU	PX692145	16,355
		X63AGU	PX692146	

### Genome sequencing and assembly

Genomic DNA of samples of *P. nobilis* (specimen vouchers: AE174PAH and Z58Mul) and *Pomacea* sp. (specimen vouchers: X60AGU and X63AGU) were sheared to reach a mean fragment length of 500 bp using a Covaris M220 Focused-ultrasonicator. The resulting fragmented DNA was used to prepare Illumina libraries using the blunt-end single-tube (BEST) protocol for double-stranded DNA [25], incorporating uniquely barcoded P5/P7 adapters during the indexing PCR. Indexing PCR was carried out in 100 µl reactions composed by 10 µl library template, 0.25 mM each dNTP, 0.25 µM each index primer (dual-barcode), 1 µl Herculase II Fusion DNA polymerase, 20 µl 5X Herculase II Reaction Buffer, and 63 µl molecular biology-grade water, under the following conditions: 95 °C for 3 min, 11 cycles of 95 °C for 20 s, 60 °C for 20 s, 72 °C for 40 s and a final extension of 72 °C for 5 min. Indexed libraries were purified using AMPure XP beads (Beckman Coulter) and diluted in 33 µl EB buffer (Qiagen). The molarity and fragment distribution of each library was measured using HS D1000 ScreenTape assay on a 4200 TapeStation System (Agilent). Equimolar pools were sent to Novogene Europe for whole genome sequencing (paired-end, 150 bp format) on the Illumina NovaSeq X platform. For the remaining specimen voucher of *P. nobilis* (AE173SHE), gDNA was sent directly to Macrogen Inc. (Seoul, South Korea) for library preparation and whole genome sequencing on the Illumina NovaSeq 6000 platform. The quality of the data was checked using FastQC 0.12.0 [26]. The number of raw read pairs per sample varied across individuals (Table 2). Raw data was trimmed and filtered using fastp v0.23.4 [27] by removing reads with > 40% low-quality bases (Phred-scaled base quality < Q15) and trimming polyG tails. The mitochondrial genome assembly was performed with GetOrganelle v1.7.7.1 [28] using the *Pomacea canaliculata* mitochondrial genome (GenBank acc. num. KU052865.1) as reference. We used Bandage v0.9.0 [29] to visualize the assembly graph. To evaluate the reliability of the mitogenome assemblies, filtered reads were mapped back to each assembled genome using BWA 0.7.17 [30]. Coverage depth across the mitochondrial genome was calculated using SAMtools 1.19.2 [31], and coverage profiles were visualized in Microsoft Excel. Tandem repeats were identified in the control region with Tandem repeats finder [32].

**Table 2:** Illumina sequencing output and average mitochondrial genome coverage

Taxon	Specimen voucher	SRA run accession	Read pairs (R1 + R2)	Total bases (Gbp)	Mean mitogenome coverage depth (×)
*Pomacea nobilis* (Reeve, 1856)	AE173SHE	SAMN56548275	160,128,700	24.0	1665.5
	AE174PAH	SAMN56548276	18,160,950	2.6	110.5
	Z58Mul	SAMN56548277	14,371,408	2.6	190.1
*Pomacea* sp.	X60AGU	SAMN56548278	21,641,640	3.2	159.7
	X63AGU	SAMN56548279	26,161,706	3.8	364.9

### Mitochondrial genome annotation

Gene annotation of the mitochondrial genome was performed with MITOS2 v2.1.0 WebServer [33] using the invertebrate genetic code. Annotation and secondary structure of transfer RNAs (tRNAs) were verified using ARWEN 1.2 [34]. Protein-coding genes (PCGs) were manually checked by detecting open reading frames with the NCBI tool ORFfinder [35]. Protein-coding genes were translated using the invertebrate mitochondrial genetic code and inspected for open reading frames. Functional annotation of the ATP6 was verified using InterProScan 5.77–108.0 (InterPro release 108.0) [36], while transmembrane regions were predicted using DeepTMHMM 2.0 [37]. The mitogenome annotation was visualized with Proksee v1.0.0a6 [38]. The ribosomal RNA (rRNA) secondary structures were inferred using R2DT 1.4 [39] and manually optimized based on the reference model of *12S rRNA* for Eukaryotes [40] and *16S rRNA* for Mollusca [41]. The potential secondary structure of the control region was predicted using RNAfold WebServer [42]. All structures were manually edited using Inkscape 1.1.

### Comparative analyses

We compared the mitogenomes of *P. nobilis* and *Pomacea* sp. with those of nine other species of the Ampullariidae family (Table 3). We calculated base composition and compositional asymmetry for five datasets: whole mitochondrial genome, control region, PCGs, rRNAs, and tRNAs. Base composition was calculated with MEGA X [43]. Compositional asymmetry was calculated using the following formulae: AT-skew = (A − T)/(A + T) and GC-skew = (G − C)/(G + C) [44]. Relative synonymous codon usage (RSCU) was calculated for each amino acid with MEGA X [43]. Nucleotide sequence similarity for each gene was calculated for all *Pomacea* mitogenomes using MEGA X [43].

### Phylogenetic analyses

A total of 34 complete mitochondrial genomes (Table 3) were included in the phylogenetic analyses, with two species of the subclass Heterobranchia, *Aplysia californica* Cooper, 1863 and *Siphonaria pectinata* (Linnaeus, 1758) as outgroups. PhyloSuite v2 [45] was used to generate, trim, and concatenate the individual alignments. The nucleotide sequences of the 13 PCGs and 2 rRNAs were aligned individually using MAFFT v7.313 [46] and trimmed with Gblocks 0.91b under relaxed conditions [47] using the following parameters: minimum number of sequences for a conserved/flank position (18/18), maximum number of contiguous non-conserved positions (8), minimum block length (10), and allowed gap positions (with half). The best-fit model and partitioning scheme were selected using the corrected Akaike Information Criterion (AICc) with PartitionFinder2 v2.1.1 [48]. Maximum likelihood (ML) and Bayesian Inference (BI) methods were used to reconstruct the phylogenetic trees on the CIPRES Science Gateway web server [49]. ML analysis was conducted in IQ-TREE 2.1.2 [50] with 10,000 ultrafast bootstrap replicates. BI analysis was performed in MrBayes 3.2 [51], with two independent runs of six Markov chain Monte Carlo (MCMC) chains running for 5 million generations, sampling every 1000 generations, and discarding the first 1.25 million generations (25%) as burn-in. Parameter convergence was assessed in Tracer v1.7 [52], ensuring all effective sample sizes (ESS) were above 200. FigTree v1.4.4 [53] was used to visualize and edit the phylogenetic trees. The final tree was formatted in Inkscape 1.1.

**Table 3:** Taxonomic classification of the taxa included in the phylogenetic analyses

Taxonomy	GenBank	Reference
Subclass Heterobranchia		
*Aplysia californica* Cooper, 1863	AY569552	[59]
*Siphonaria pectinata* (Linnaeus, 1758)	AY345049	[18]
Subclass Caenogastropoda		
Unassigned Order, Superfamiliy Cerithioidea		
*Turritella bacillum* Kiener, 1843	KU221394	[60]
*Melanoides tuberculata* (O. F. Müller, 1774)	MZ321058	[61]
Order Littorinomorpha		
*Littorina saxatilis* (Olivi, 1792)	KU952094	[62]
*Aliger gigas* (Linnaeus, 1758)	KM245630	[63]
*Polinices sagamiensis* Pilsbry, 1904	MK478018	[64]
*Cypraea tigris* Linnaeus, 1758	MK783263	[65]
Order Neogastropoda		
*Cymbium olla* (Linnaeus, 1758)	EU827199	[66]
*Columbella adansoni* Menke, 1853	KP716637	[67]
*Nassarius gregarius* (Grabau & King, 1928)	MT075611	[68]
*Rapana venosa* (Valenciennes, 1846)	EU170053	Chandler et al. (unpubl.)
*Conus quercinus* [Lightfoot], 1786	KY609509	[69]
“Order” Architaenioglossa		
Superfamily Cyclophoroidea		
Family Cyclophoridae		
*Cyclophorus martensianus* Möllendorff, 1874	MH700493	[70]
Family Cochlostomatidae		
*Obscurella hidalgoi* (Crosse, 1864)	KP716638	[67]
Superfamily Viviparoidea		
Family Viviparidae		
*Cipangopaludina catayensis* (Heude, 1890)	KM503121	[71]
*Dalipaludina oxytropoides* (Heude, 1889)	KY200976	[72]
*Rivularia auriculata* (E. von Martens, 1875)	MN264502	[73]
*Sinotaia quadrata* (W. H. Benson, 1842)	KX688548	Mu et al. (unpubl.)
*Viviparus chui* Yen, 1937	KY679829	[72]
Superfamily Ampullarioidea		
Family Ampullariidae		
*Marisa cornuarietis* (Linnaeus, 1758)	KM100140	[74]
*Pila globosa* (Swainson, 1822)	OQ682616	Ayyagari (unpubl.)
*Pila virens* (Lamarck, 1822)	OQ131126	Ayyagari (unpubl.)
*Pomacea canaliculata* (Lamarck, 1822)	KJ739609	[75]
*Pomacea maculata* Perry, 1810	MF401379	[76]
*Pomacea occulta* Yang & Yu, 2019	KR350466	[77]
*Pomacea diffusa* Blume, 1957	MF373586	[78]
*Pomacea aulanieri* (Deville & Hupé, 1850)	OR253803	[21]
*Pomacea reevei* Ampuero & Ramírez, 2023	OR253802	[21]

### Selection pressure analyses

The non-synonymous (*K*_*a*_) and synonymous (*K*_*s*_) substitution rates of the 13 protein-coding genes were estimated using DnaSP v6 [54]. Evidence for episodic positive selection was tested with BUSTED [55] implemented in the Datamonkey Adaptive Evolution Server [56]. An initial screening was conducted using a concatenated dataset of the 13 PCGs across all available Ampullariidae mitogenomes to identify putative clade-specific signals of selection. Subsequently, gene-specific analyses were performed to pinpoint the contribution of individual genes, focusing on the clade that showed evidence of selection in the initial screening. For the remaining clades, only the genes identified in this step were further evaluated. Site-level selection was assessed with FEL [57], and changes in the intensity of selection were analyzed using RELAX [58].

## Results

### Mitogenome size and structure

**Fig. 1:**
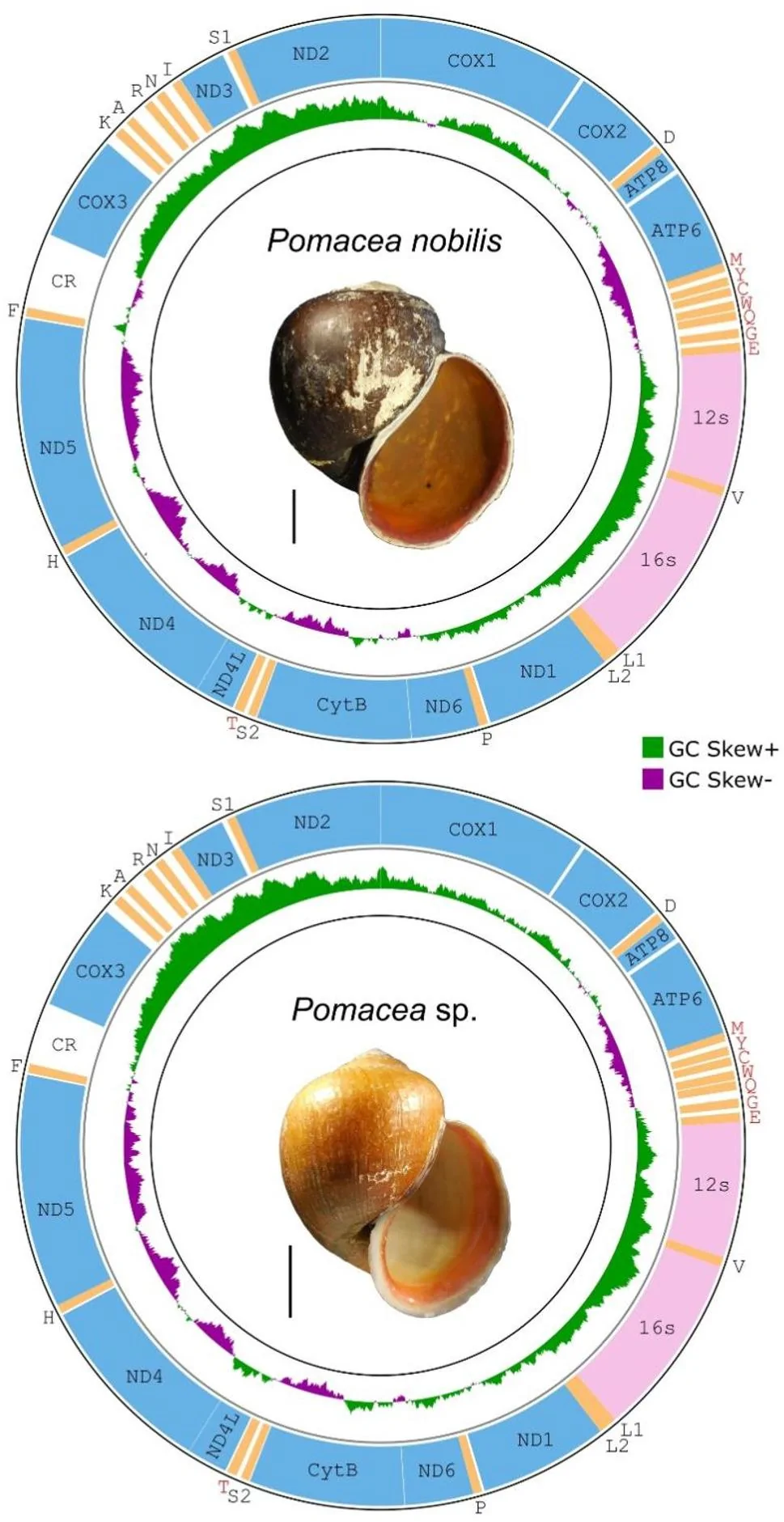
Mitochondrial genome of *Pomacea nobilis* and *Pomacea* sp. Different gene types are shown in different colors: Blue for PCGs, Violet for ribosomal RNA and Orange for tRNAs. Gene names are color coded by H (black) or L (red) strand. tRNA genes were named with their corresponding amino acid using the IUPAC single letter codes. The inner circle shows the GC skew. Shell height: 99.4 mm for *P. nobilis* and 74.8 mm for *Pomacea* sp. Scale bar: 20 mm

The three mitochondrial genomes of *P. nobilis* ranged from 16,279 to 16,471 bp, whereas the two *Pomacea* sp. mitogenomes were identical in sequence and length (16355 bp) (Table 1). Each mitogenome contained the typical set of 37 genes (13 PCGs, 2 rRNAs, and 22 tRNAs) plus a control region (CR) (Fig. 1). Twenty-nine genes were encoded on the heavy strand (H), while eight tRNA genes were encoded on the light strand (L) (Fig. 1; Table 4). On average, the PCGs, rRNAs, tRNAs and CR comprised 68.99%, 14.35%, 9.08%, and 3.04% of the mitochondrial genome of *P. nobilis*, respectively. Very similar values were found in *Pomacea* sp. (PCGs = 69.19%, rRNAs = 14.37%, tRNAs = 9.10%, CR = 2.78%). The proportion of intergenic regions was very similar among the assembled mitogenomes, representing 4.80–4.86% (791 bp in average) of the mitogenome in *P. nobilis* and 4.57% (747 bp) in *Pomacea* sp. In contrast, the assembled control region was more variable, ranging from 375 to 567 bp among the three *P. nobilis* individuals, whereas both *Pomacea* sp. specimens exhibited a length of 455 bp.

**Table 4:** Mitochondrial genome annotation for *Pomacea nobilis* and *Pomacea* sp. When values differ between taxa, the annotation for *P. nobilis* is shown on the left, and the annotation for *Pomacea* sp. is shown on the right

Gene	Strand	Length (bp)	Amino acid length	Start codon	Stop codon	Anti-codon	Intergenic length (bp)
*COI*	H	1536	511	ATG	TAA	-	28
*COII*	H	687	228	ATG	TAA	-	19
*tRNA-Asp*	H	68	-	-	-	GTC	2 / 3
*ATP8*	H	156	51	ATG	TAA	-	34
*ATP6*	H	768	260	ATG	TAA	-	-39
*tRNA-Met*	L	65 / 64	-	-	-	CAT	39
*tRNA-Tyr*	L	65 / 64	-	-	-	GTA	15 / 16
*tRNA-Cys*	L	63	-	-	-	GCA	37
*tRNA-Trp*	L	67	-	-	-	TCA	10 / 9
*tRNA-Gln*	L	66	-	-	-	TTG	62
*tRNA-Gly*	L	68	-	-	-	TCC	34
*tRNA-Glu*	L	68	-	-	-	TTC	0
*12S rRNA*	H	996 / 994	-	-	-	-	0
*tRNA-Val*	H	71	-	-	-	TAC	0
*16S rRNA*	H	1357 / 1356	-	-	-	-	0
*tRNA-Leu1*	H	66	-	-	-	TAG	0
*tRNA-Leu2*	H	68	-	-	-	TAA	0
*NAD1*	H	945	314	ATG	TAA	-	19
*tRNA-Pro*	H	67	-	-	-	TGG	0
*NAD6*	H	498	165	ATG	TAA	-	5
*CytB*	H	1140	379	ATG	TAG	-	8
*tRNA-Ser2*	H	63	-	-	-	TGA	33 / 34
*tRNA-Thr*	L	74	-	-	-	TGT	14
*NAD4L*	H	297	98	ATG	TAA	-	-7
*NAD4*	H	1362	453	ATG	TAA	-	16
*tRNA-His*	H	66 / 65	-	-	-	GTG	0
*NAD5*	H	1710	569	ATG	TAA	-	14
*tRNA-Phe*	H	68	-	-	-	GAA	0
*CR*	H	375–567 / 455	-	-	-	-	0
*COIII*	H	786	261	ATG	TAA	-	80 / 81
*tRNA-Lys*	H	73	-	-	-	TTT	46
*tRNA-Ala*	H	67	-	-	-	TGC	111
*tRNA-Arg*	H	68 / 69	-	-	-	TCG	43 / 42
*tRNA-Asn*	H	71	-	-	-	GTT	74
*tRNA-Ile*	H	70	-	-	-	GAT	3
*NAD3*	H	354	117	ATG	TAA	-	41 / 40
*tRNA-Ser1*	H	68	-	-	-	GCT	0
*NAD2*	H	1077	358	ATG	TAA	-	4

Within *Pomacea*, intergenic regions represented an average of 2.55% of the mitogenome in the *P. canaliculata* clade (*P. canaliculata*: 2.43%; *P. maculata*: 2.44%; *P. occulta*: 2.64%; *P. reevei*: 2.69%) and 3.39% in the *P. bridgesii* clade (*P. diffusa*: 3.32%; *P. aulanieri*: 3.46%). When compared with the additional Ampullariidae included in our dataset, higher proportions of intergenic regions were observed in *Marisa cornuarietis* (5.38%), *Pila globosa* (6.81%), and *Pila virens* (7.06%). Aside from the control region, the largest intergenic region in both *P. nobilis* and *Pomacea* sp. (111 bp) was located between *tRNA-Ala* and *tRNA-Arg* (Table 4). Additional non-coding regions with comparable lengths in both lineages were found between *COIII/tRNA-Lys* (80–81 bp), *tRNA-Asn/tRNA-Ile* (74 bp), and *tRNA-Gln/tRNA-Gly* (62 bp). The two lineages also shared the same overlapping regions: *ATP6/tRNA-Met* (39 bp) and *NAD4L/NAD4* (7 bp).

### Mitochondrial base composition and protein-coding gene features

The overall base composition of the mitochondrial genome of *P. nobilis* and *Pomacea* sp. is very similar and aligns with the base composition of other Ampullariidae mitogenomes, although *P. nobilis* displays the highest AT-skew (followed by *Pomacea* sp.) and the lowest GC-skew in the family (Table 5). On average, species of the *Effusa* clade exhibit slightly higher A + T content (69.27–69.64%) than those in the *P. bridgesii* clade (68.85–69.49%), but lower than species in the *P. canaliculata* clade (70.98–72.31%). The A + T content, AT-skew and GC-skew for the PCGs, rRNAs, tRNAs and CR of *P. nobilis* and *Pomacea* sp. are consistent with values observed in other Ampullariidae (Table 6), although the percentage of A + T content varies according to the annotated feature in both lineages, being highest in the control region and lowest for the PCGs (CR> rRNAs> tRNAs> PCGs).

**Table 5:** Nucleotide composition and skew statistics for Ampullariidae mitogenomes excluding the control region. For *Pomacea nobilis*, values represent averages from three sequences

Taxon	Size (bp)	A%	T%	G%	C%	A + T%	G + C%	AT skew	GC skew
*Pila globosa*	16,318	29.56	41.55	16.57	12.32	71.11	28.89	-0.169	0.147
*Pila virens*	16,369	27.11	41.82	19.38	11.69	68.93	31.07	-0.214	0.248
*M. cornuarietis*	15,860	28.95	41.03	16.70	13.33	69.97	30.03	-0.173	0.112
*P. canaliculata*	15,348	30.27	41.24	15.67	12.82	71.51	28.49	-0.153	0.100
*P. maculatta*	15,363	30.76	41.15	15.32	12.77	71.91	28.09	-0.145	0.091
*P. occulta*	15,373	30.70	41.10	15.33	12.87	71.80	28.20	-0.145	0.087
*P. reevei*	15,368	29.98	40.88	15.92	13.22	70.86	29.14	-0.154	0.093
*P. aulanieri*	15,572	29.35	39.41	16.47	14.77	68.76	31.24	-0.146	0.054
*P. diffusa*	15,567	29.50	39.78	16.37	14.36	69.27	30.73	-0.148	0.065
*Pomacea nobilis*	15,904	30.13	38.97	16.15	14.76	69.09	30.91	-0.128	0.045
*Pomacea* sp.	15,900	30.19	39.19	16.11	14.52	69.38	30.62	-0.130	0.052

**Table 6:** Nucleotide content and skew statistics for major gene categories of Ampullariidae mitogenomes. For *Pomacea nobilis*, values represent averages from three sequences

Taxon	PCGs			rRNAs			tRNAs			CR		
	A + T%	AT skew	GC Skew	A + T%	AT skew	GC Skew	A + T%	AT skew	GC skew	A + T%	AT skew	GC Skew
*Pila globosa*	69.73	-0.22	0.13	72.32	-0.01	0.21	71.96	-0.03	0.18	74.32	-0.03	0.17
*Pila virens*	67.44	-0.28	0.21	70.94	-0.07	0.33	71.67	-0.01	0.18	63.40	-0.10	0.49
*M. cornuarietis*	68.98	-0.23	0.08	71.53	-0.03	0.24	68.07	0.02	0.13	71.43	-0.24	-0.11
*P. canaliculata*	70.69	-0.21	0.09	72.87	0.01	0.19	71.95	-0.03	0.08	79.39	0.26	0.41
*P. maculatta*	71.14	-0.20	0.07	73.11	0.01	0.20	71.92	-0.03	0.09	86.57	0.02	0.00
*P. occulta*	70.96	-0.14	0.09	73.02	0.02	0.18	71.97	-0.03	0.09	85.82	-0.01	0.00
*P. reevei*	69.98	-0.21	0.07	72.63	0.03	0.18	71.81	0.00	0.15	77.40	-0.05	0.18
*P. aulanieri*	67.57	-0.20	0.03	71.58	0.03	0.16	69.63	-0.02	0.15	71.56	-0.07	-0.17
*P. diffusa*	67.98	-0.21	0.05	73.11	0.01	0.20	71.57	-0.02	0.06	73.70	0.03	-0.06
*Pomacea nobilis*	67.98	-0.19	0.02	70.96	0.04	0.15	70.29	0.00	0.14	75.08	-0.10	-0.03
*Pomacea sp.*	68.13	-0.20	0.02	71.45	0.04	0.15	69.74	-0.01	0.15	78.68	-0.12	0.01

**Fig. 2:**
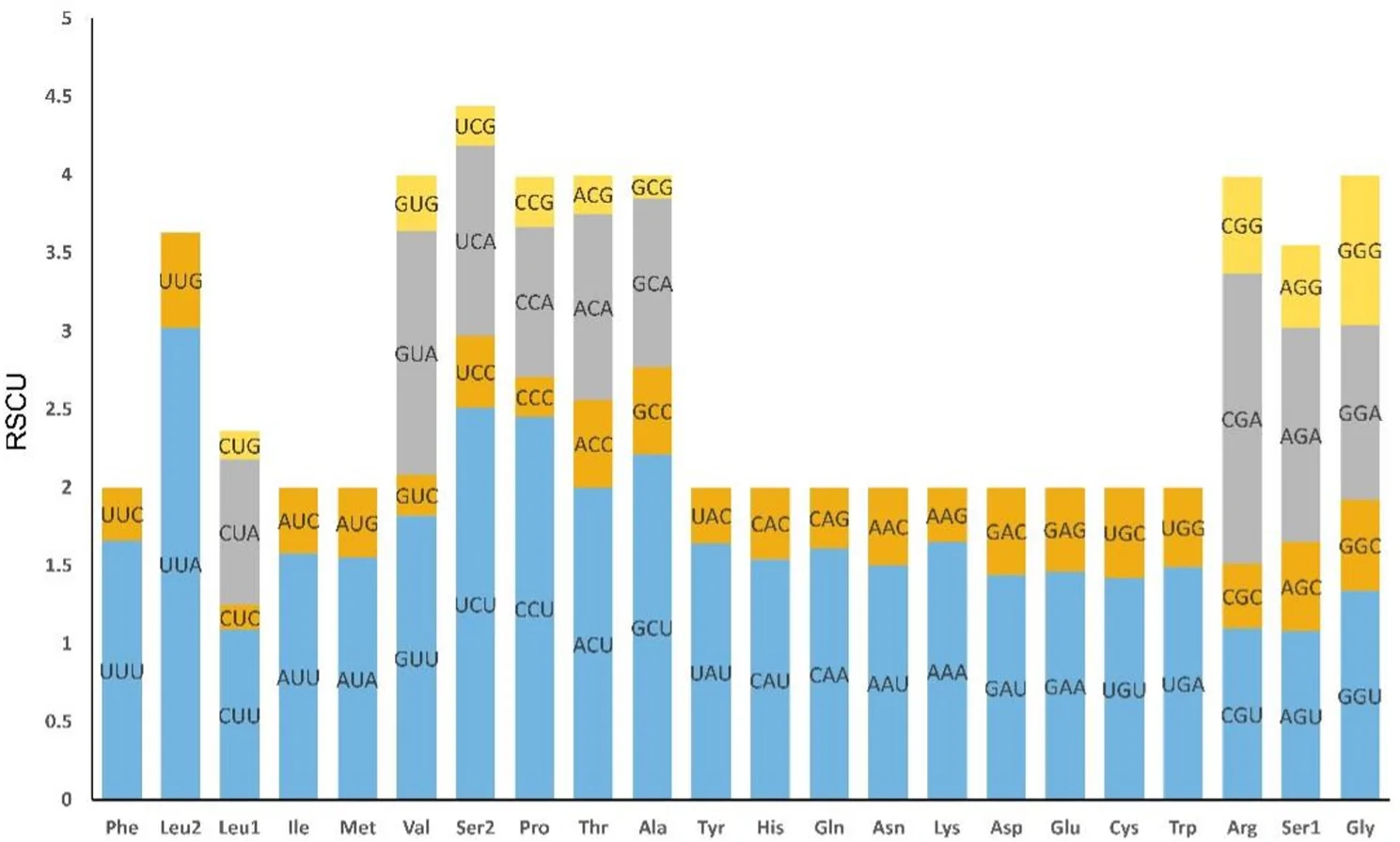
Relative synonymous codon usage (RSCU) of the mitogenome of *P. nobilis*

Among the 13 PCGs, *NAD5* is the longest (1710 bp) and *ATP8* the shortest (156 bp) in both lineages. When compared with additional mitogenomes in our dataset, only five genes (*COI*, *COII*, *CytB*, *NAD4L*, and *NAD3*) maintain the same length across all *Pomacea* species. In contrast, *ATP8*, *ATP6*, *NAD6*, *COIII*, and *NAD2* show lengths unique to *P. nobilis* and *Pomacea* sp. Notably, ATP6 was annotated as 768 bp in both lineages, exceeding the lengths observed in other available ampullariid mitogenomes. Alignment of nucleotide and amino acid sequences indicated that this difference results from a 3′ extension shared by the sampled representatives of the *Effusa* clade (Additional files 2 and 3). Among the remaining genes, *NAD1* and *NAD4* in both species match the lengths observed in the *P. bridgesii* clade, whereas *NAD5* aligns with that of the *P. canaliculata* clade. While all PCGs begin with the typical ATG codon, twelve of the 13 PCGs end with TAA, except for *CytB*, which uses TAG as stop codon. The analysis of the relative synonymous codon usage (RSCU) in *P. nobilis* (Fig. 2) and *Pomacea* sp. (Additional file 4) shows that UUA (Leu), UUU (Leu) and AUU (Ile) are the three most commonly used codons. On the other hand, the least frequently used codon is CGC (Arg), followed closely by CGG (Arg), CCC (Pro) and GCG (Ala) in *P. nobilis*; and CCG (Pro) and CGG (Arg) in *Pomacea* sp. Overall, codons ending in A or T are predominant in both species (78.7%).

### Mitochondrial rRNAs, tRNAs, and control region features

**Fig. 3:**
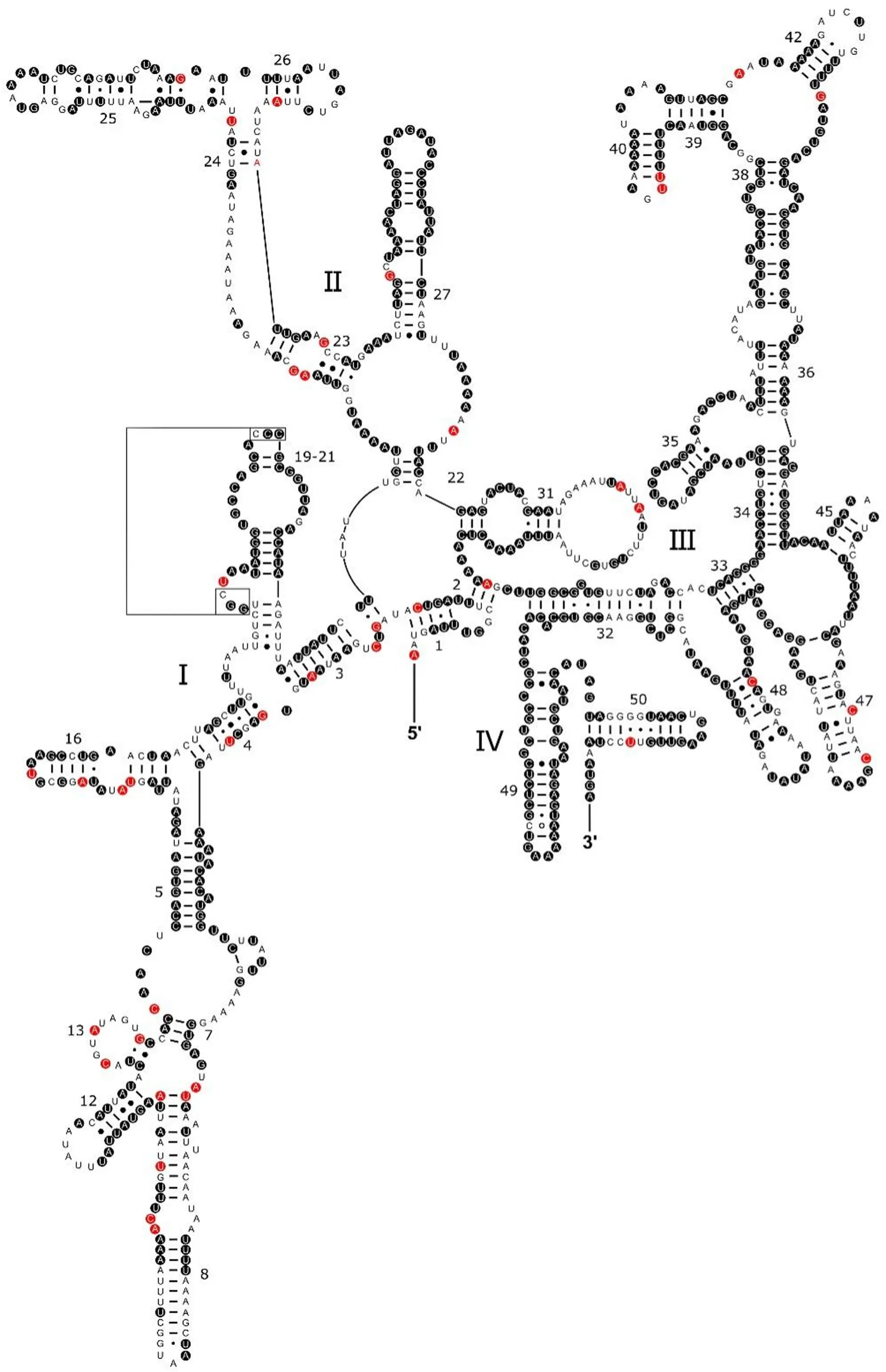
Secondary structure of the *12S rRNA* in *Pomacea nobilis* and *Pomacea* sp. Conserved sites within *Pomacea* are marked in black, while variable positions between *P. nobilis* and *Pomacea* sp. are marked in red. Intraspecific variation within *P. nobilis* or within other *Pomacea* species is not highlighted

**Fig. 4:**
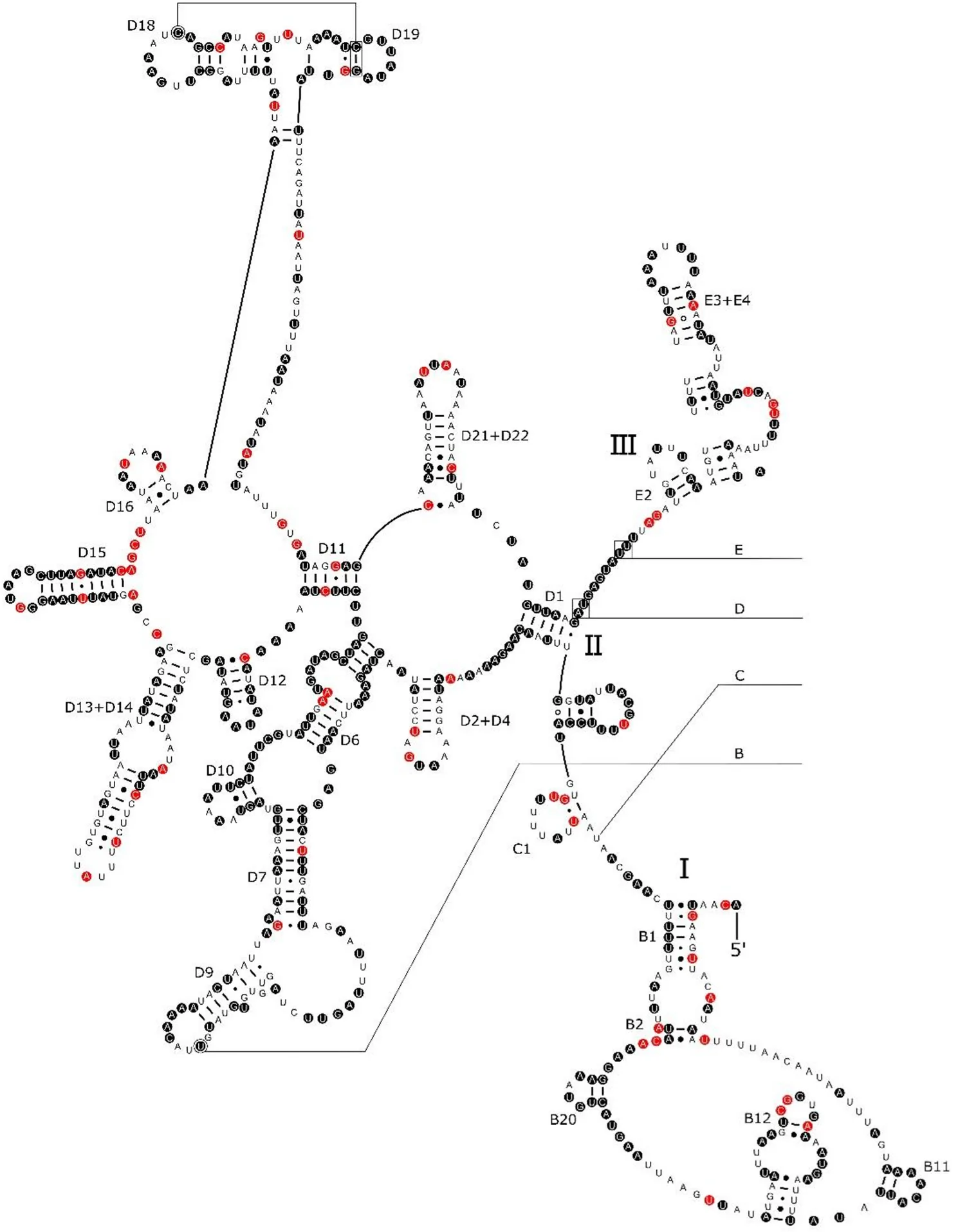
Secondary structure of the Domains I-III of the *16S rRNA* in *Pomacea nobilis* and *Pomacea* sp. Conserved sites within *Pomacea* are marked in black, while variable positions between *P. nobilis* and *Pomacea* sp. are marked in red. Intraspecific variation within *P. nobilis* or within other *Pomacea* species is not highlighted

**Fig. 5:**
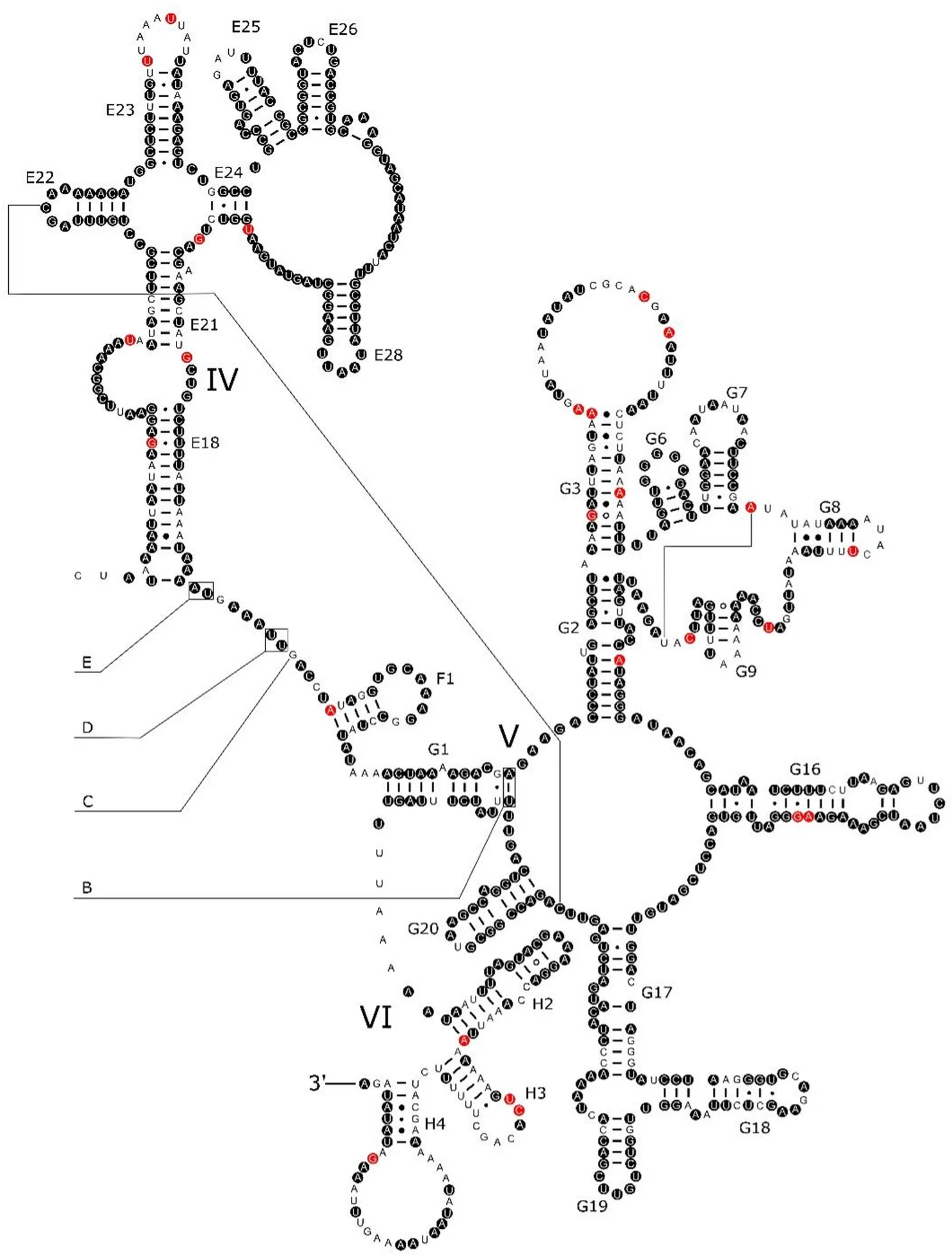
Secondary structure of the Domains IV-VI of the *16S rRNA* in *Pomacea nobilis* and *Pomacea* sp. Conserved sites within *Pomacea* are marked in black, while variable positions between *P. nobilis* and *Pomacea* sp. are marked in red. Intraspecific variation within *P. nobilis* or within other *Pomacea* species is not highlighted

The *12S rRNA* (994–996 bp) and *16S rRNA* (1356–1357 bp) of *P. nobilis* and *Pomacea* sp. are the largest recorded for *Pomacea*, exceeding those observed in the species of the *P. canaliculata* clade (909–936 bp for *12S*, 1331–1340 bp for *16S*) and *P. bridgesii* clade (952–960 bp for *12S*, 1346–1348 bp for *16S*), although smaller than in *Pila* (1012–1017 bp for *12S*, 1417–1423 bp for *16S*). The alignment of all available complete *12S rRNA* sequences of *Pomacea* resulted in a length of 1002 bp, with 615 conserved sites (61.38%). The predicted secondary structure comprises four domains (I–IV), with Domains III (71.58% of invariant sites) and IV (85.14% of invariant sites) being the most conserved, whereas some regions in the Domains I and II showed high variability, such as stems 8, 24 and 25, and loops 13, 31 and 42 (Fig. 3). Some differences were also observed between *P. nobilis* and *Pomacea* sp., especially in stems 8, 13 and 16 of Domain I and stem 23 of Domain II. The alignment of all available complete *16S rRNA* sequences of *Pomacea* resulted in a length of 1385 bp, with 853 conserved sites (61.59%). The predicted secondary structure comprises six domains (I–VI), with Domains IV (80.28% of invariant sites) and V (73.44% of invariant sites) being the most conserved, whereas stem-loops D2 + D4, D13 + D14, D21 + D22, E3 + E4 and H3 show a larger proportion of variable sites, with small size differences in some loops, particularly in stem-loops D13 + D14 and H4 (Figs. 4 and 5).

**Fig. 6:**
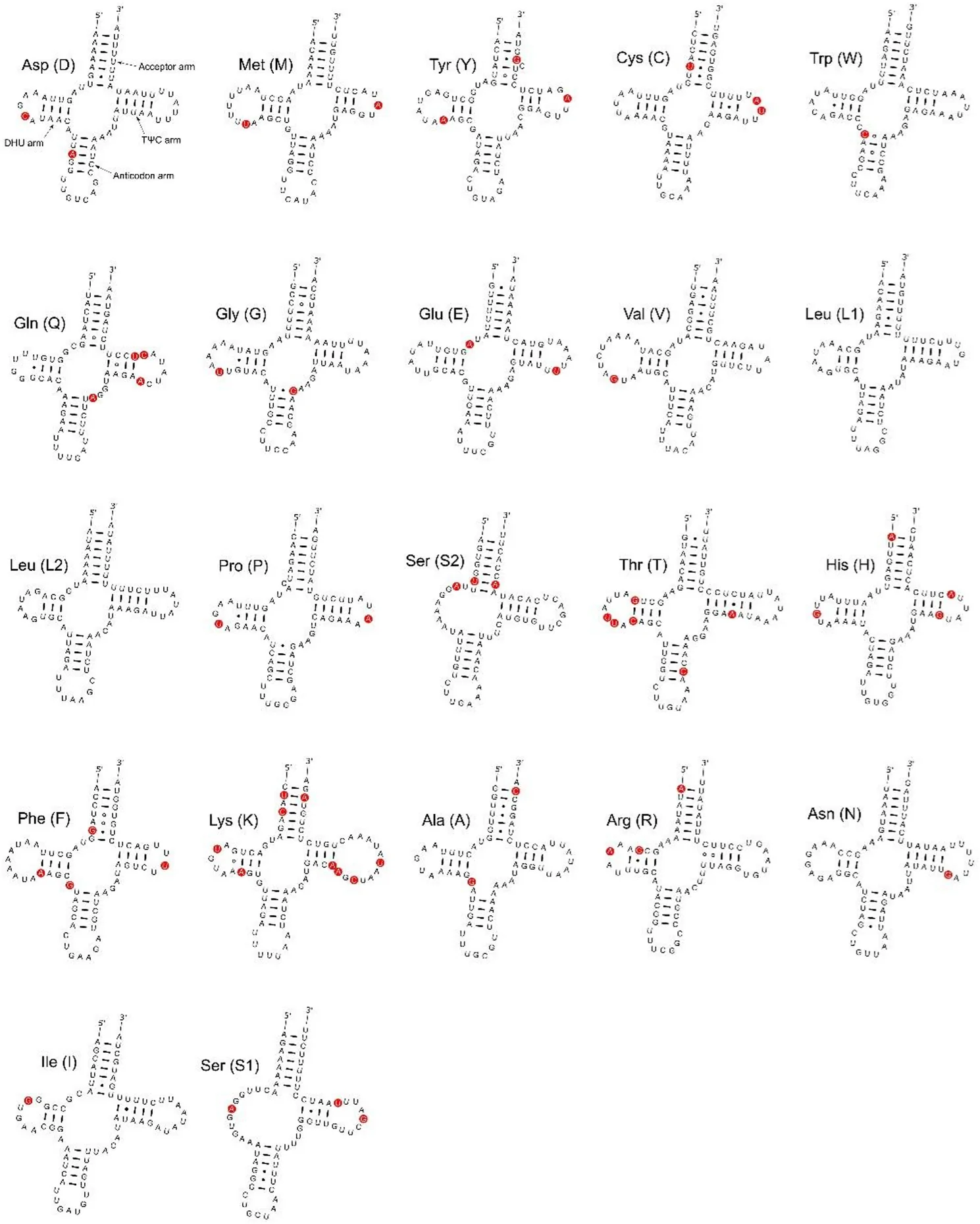
Shared secondary structure of the tRNAs in the mitogenomes of *Pomacea nobilis* and *Pomacea* sp. Variable sites between both lineages are marked in red

The tRNA genes in both *P. nobilis* and *Pomacea* sp. ranged from 63 bp (*tRNA-Cys* and *tRNA-Ser*) to 74 bp (*tRNA-Thr*). A comparison of the secondary structure of the 22 tRNAs in both species with those of other *Pomacea* showed that the typical clover-leaf structure with four stem-loops is conserved in most tRNAs (Fig. 6), although slight differences in the size of the D and T loops were observed in some tRNAs (e.g. *tRNA-Gln* and *tRNA-Ala)*. The only exceptions are the tRNA genes for serine, in which the D-arm can be replaced by a large bulge. This modified structure occurs in *tRNA-Ser1* in *Pila*, *Marisa*, and most species of the *P. canaliculata* clade, but not in the *P. bridgesii* clade, where *tRNA-Ser2* is the modified tRNA. In *P. nobilis* and *Pomacea* sp., both tRNAs for serine exhibit the modified structure (Fig. 6).

**Fig. 7:**
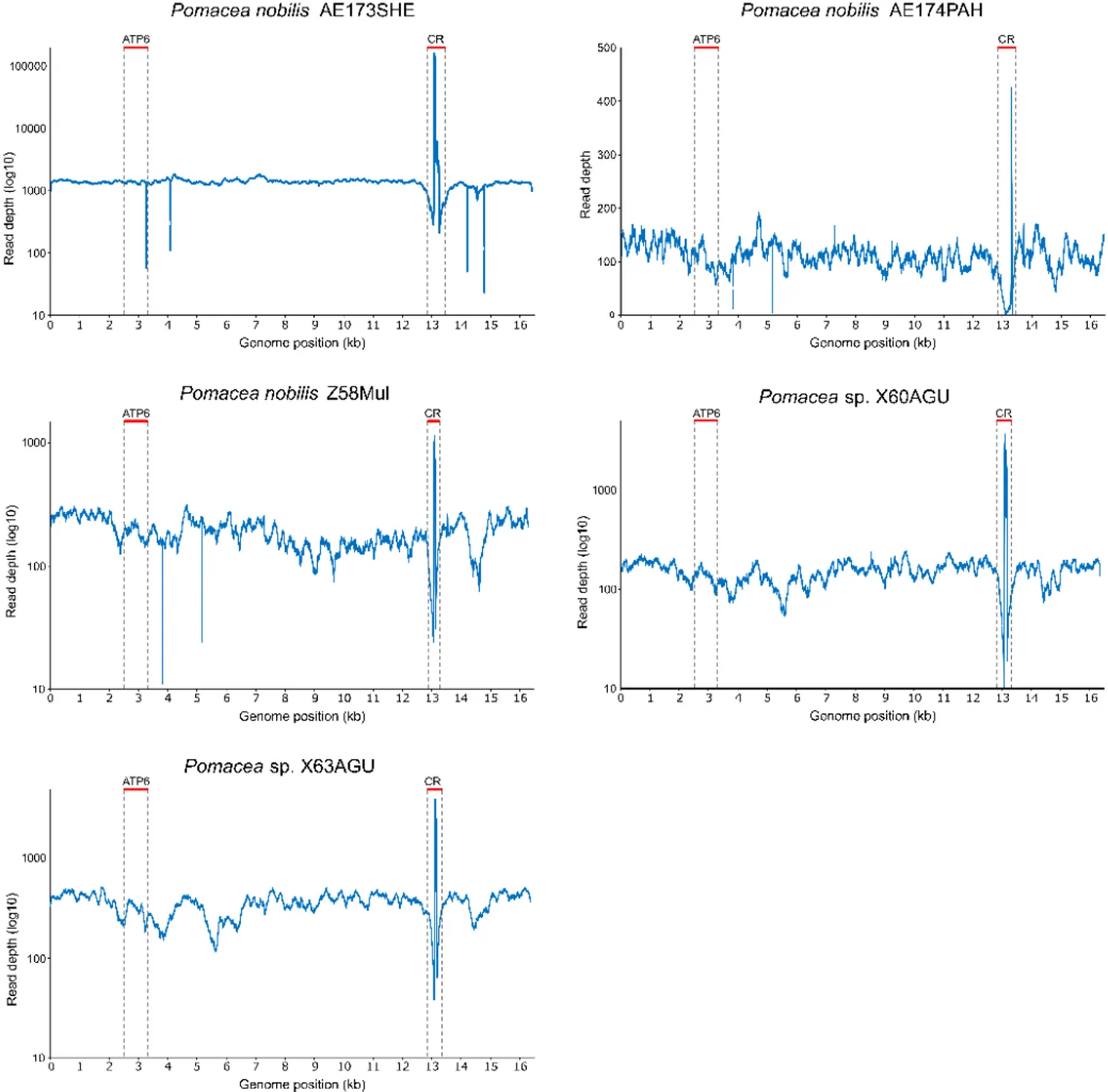
Read coverage across the assembled mitogenomes of *P. nobilis* and *Pomacea* sp. Segments corresponding to the control region (CR) and ATP6 gene are highlighted

The length range of the assembled control region in *P. nobilis* (375–567 bp) and *Pomacea* sp. (455 bp) falls between the values previously reported for the *P. bridgesii* (524–806 bp) and *P. canaliculata* clades (141–433 bp). This region showed a high AT content and contained several AT-rich tracts, with tandem repeat analysis suggesting different candidate repeat motifs among the mitogenomes analyzed (Table 7). Read coverage was generally uniform across most of the mitogenome but showed a pronounced peak in the control region in all samples (Fig. 7).

**Table 7:** Tandem repeats identified in the mitochondrial control region

Taxon	Specimen voucher	Control region length (bp)	Repeat motif	Repeat type	Repeat length (bp)
*Pomacea nobilis*	AE173SHE	555	AT(n)	imperfect microsatellite	130
	AE174PAH	567	CTCTCTTTCTCT	imperfect tandem repeat	57
	Z58Mul	375	(TA)n	perfect microsatellite	41
*Pomacea* sp.	X60AGU/ X63AGU	455	(TA)n	imperfect microsatellite	81

**Fig. 8:**
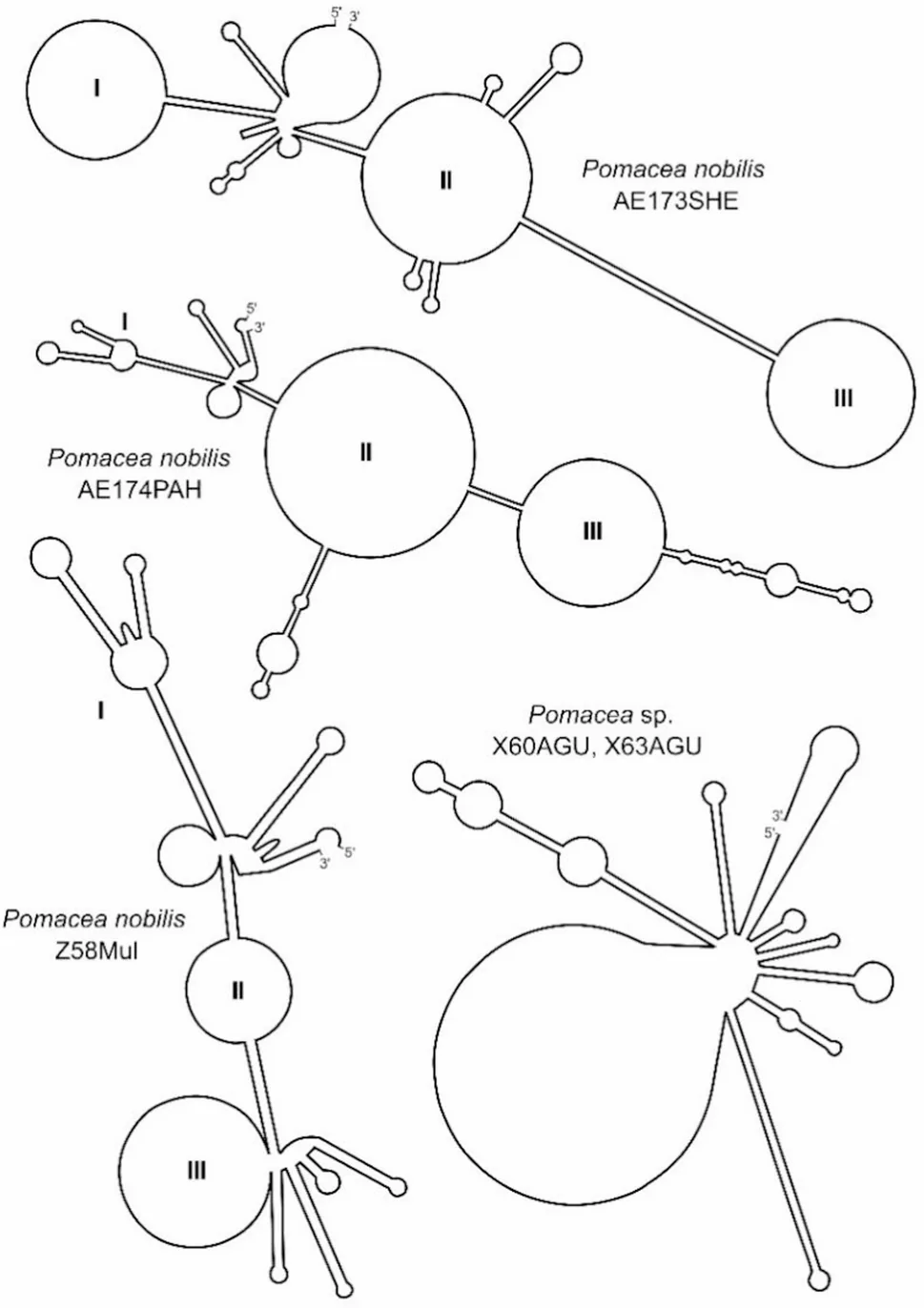
Predicted secondary structure of the mitochondrial control region of *Pomacea nobilis* (specimen vouchers: AE173SHE, AE174PAH, and Z58Mul) and *Pomacea* sp. (specimen vouchers: X60AGU and X63AGU)

In *P. nobilis*, the predicted secondary structure of the control region (Fig. 8) suggests the presence of three main loops (I–III) and several smaller stem-loop structures, with marked differences in the development of Loop I and in the number and size of stem-loops between Loops I and II among the three mitogenomes analyzed. In contrast, the predicted secondary structure of the assembled control region of *Pomacea* sp. showed a different organization with a putative large central loop surrounded by multiple stem-loop structures (Fig. 8).

### Phylogenetic analysis and genetic distances

**Fig. 9:**
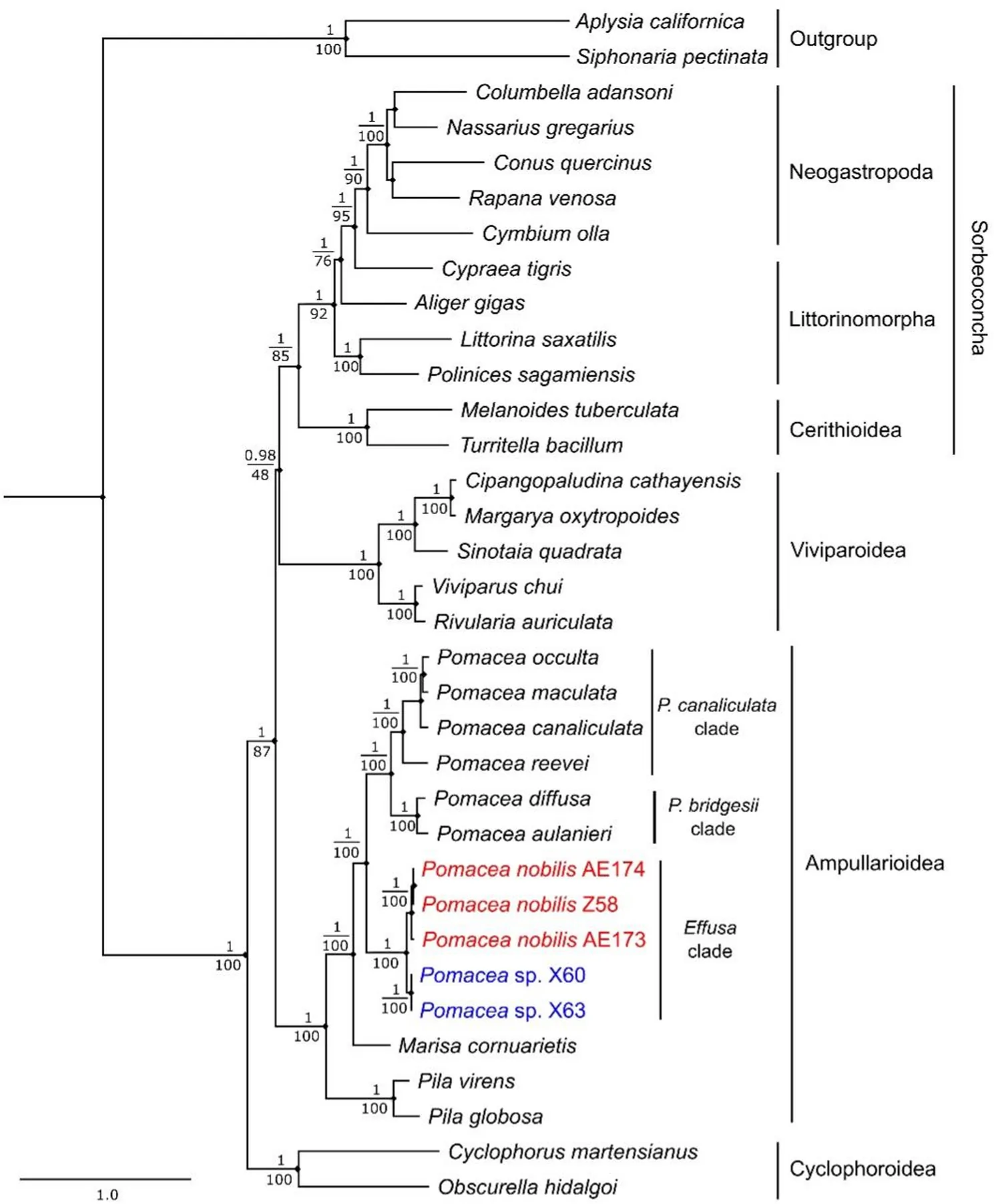
Phylogenetic relationships of *Pomacea* and other Caenogastropoda species based on the nucleotide sequences of 13 PCGs and 2 rRNAs. The Bayesian inference (BI) tree is shown. Posterior probabilities (BI) and bootstrap support values (%) from the Maximum likelihood (ML) analysis are shown above and below the branches, respectively. Samples sequenced in this study are shown in color red (*P. nobilis*) and blue (*Pomacea* sp.). The scale represents the number of substitutions per site

Maximum likelihood (ML) and Bayesian inference (BI) phylogenetic analyses produced the same topology (Fig. 9), although the BI showed higher support values (0.98–1.00) than the ML (48–100). Viviparoidea, Ampullarioidea, and Cyclophoroidea were each recovered as monophyletic superfamilies. Viviparoidea was recovered as the sister lineage to Sorbeoconcha, although support for this relationship was inconsistent between analyses (BI: 0.98, ML: 48), indicating uncertainty in deeper caenogastropod relationships. Relationships within Ampullariidae were well-supported: the *P. canaliculata* and *P. bridgesii* clades were recovered as monophyletic groups, and the *Effusa* clade (*P. nobilis* + *Pomacea* sp.) formed a strongly supported sister group to both clades (BI: 1.00, ML: 100). Based on the available whole mitogenomes, the observed intraspecific variation in *P. nobilis* was 2.58% among samples from Loreto, whereas the divergence between *P. nobilis* and *Pomacea* sp. was 7.23%.

**Fig. 10:**
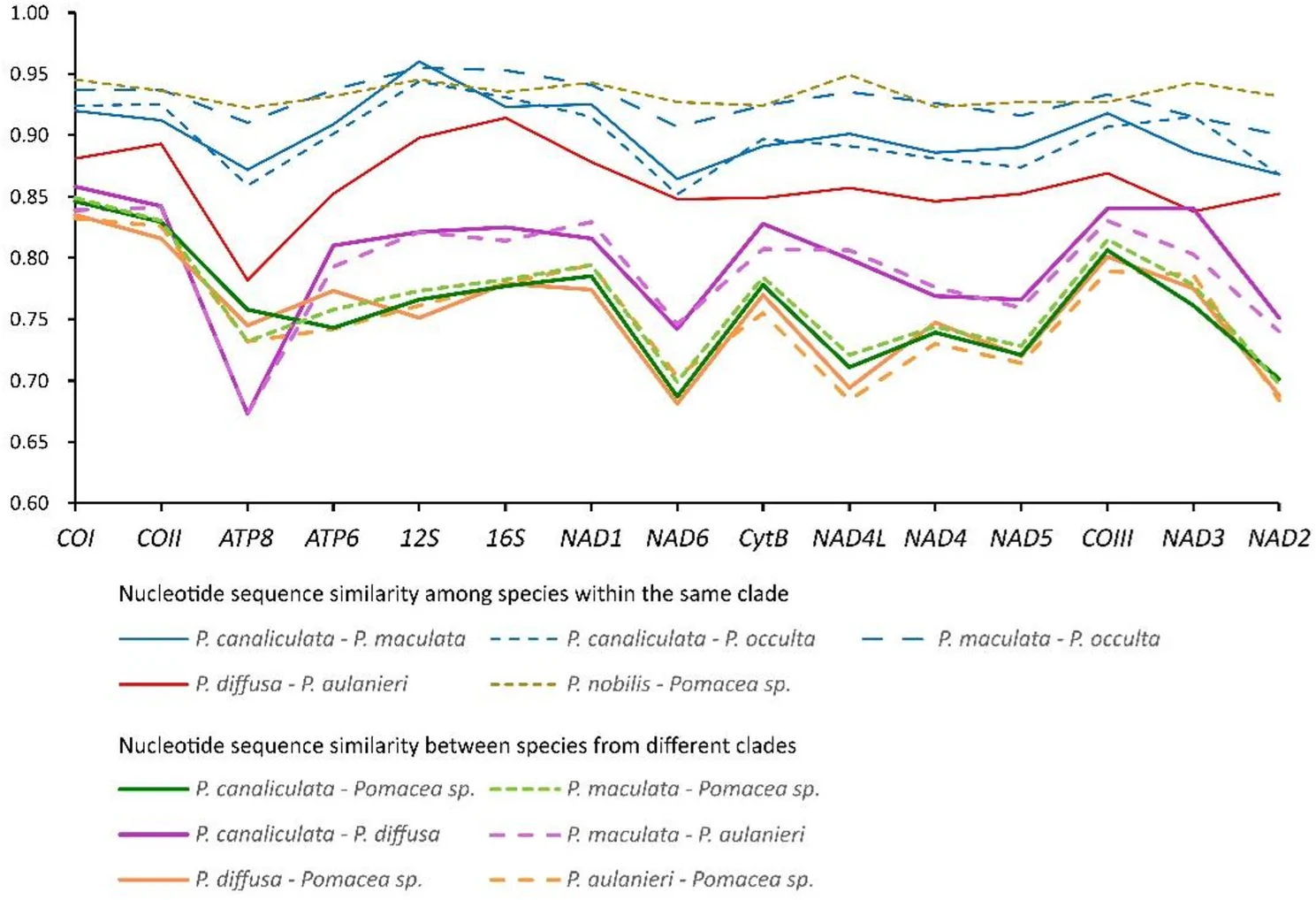
Pairwise nucleotide sequence similarity of the 13 PCGs and 2 rRNAs among the three *Pomacea* clades: *P. canaliculata* group (*P. canaliculata*, *P. maculata*, and *P. occulta*), *P. bridgesii* group (*P. diffusa* and *P. aulanieri*), and *Effusa* group (*P. nobilis* and *Pomacea* sp.)

A comparison of the nucleotide sequence similarity among *Pomacea* mitogenomes (Fig. 10) showed consistent higher similarities among species within the same clade (*P. canaliculata*,* P. bridgesii*, and *Effusa*), than between species from different clades. This analysis also supports that species from *P. canaliculata* group are more similar to *P. bridgesii* species than to the *Effusa* clade, which is consistent with the phylogenetic tree (Fig. 9). This comparison also indicated that the least conserved genes are *NAD6*, *NAD2* and *ATP8*, suggesting their potential utility for population-level genetic studies.

### Selection pressure analyses

**Fig. 11:**
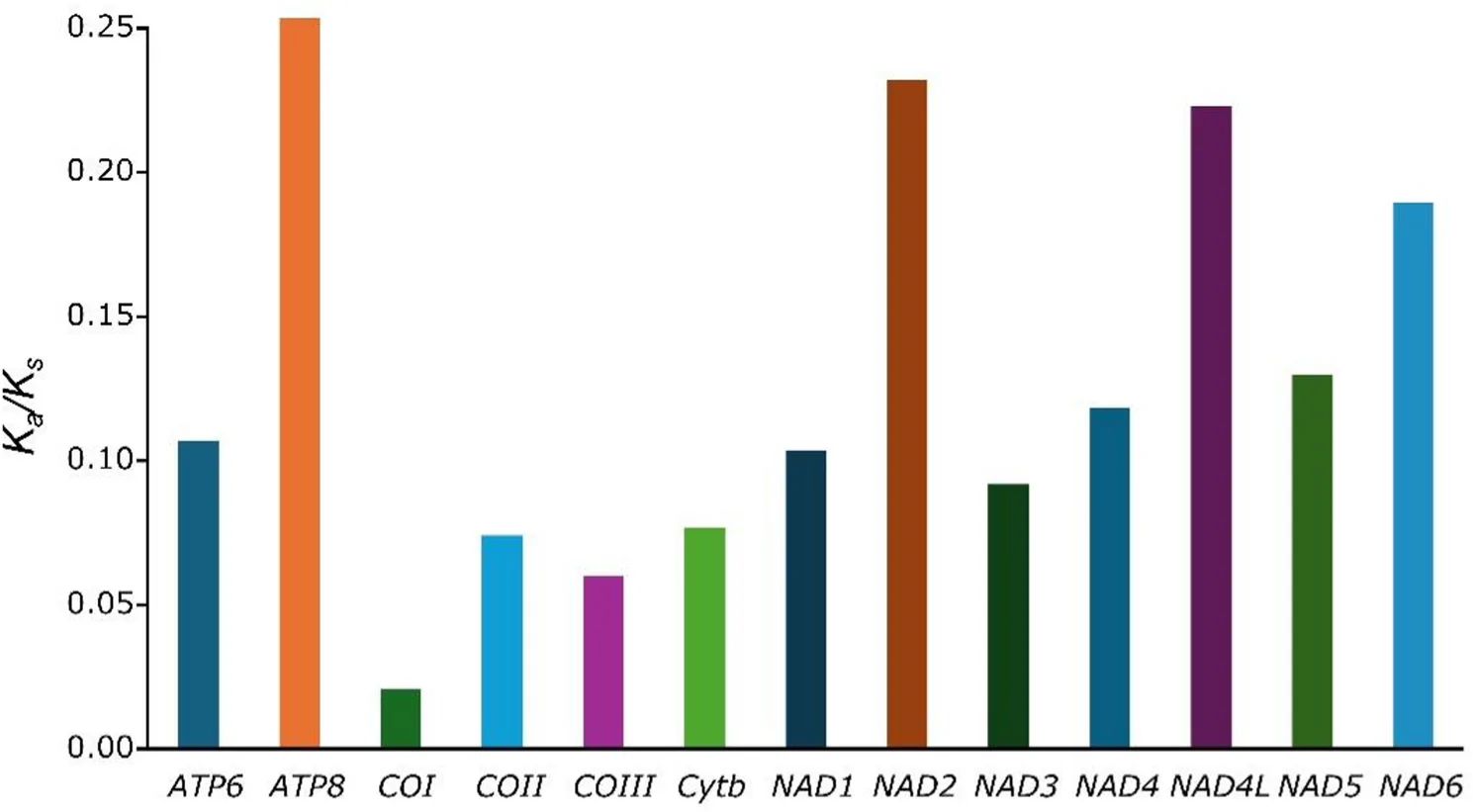
Rates of non-synonymous (*K*_*a*_) over synonymous (*K*_*s*_) substitution rates of the 13 protein-coding genes in *Pomacea*

The *K*_*a*_*/K*_*s*_ ratios calculated for the 13 PCGs were all below 1, reflecting overall low rates of nonsynonymous substitutions across the mitochondrial genome of *Pomacea* (Fig. 11). The highest ratios were observed in *ATP8* (0.253), *NAD2* (0.232), and *NAD4L* (0.223), whereas the cytochrome oxidase genes showed comparatively lower values, indicating variation in evolutionary rates among genes. No significant signal of positive selection was detected in BUSTED for most genes (*p* > 0.05). However, the gene encoding cytochrome c oxidase subunit III (*COIII*) in the *P. bridgesii* clade showed evidence of episodic diversifying selection (*p* = 0.025), although this signal was not significant after multiple testing correction using the Benjamini-Hochberg procedure (FDR-adjusted *p* = 0.076). Site-level analysis with FEL identified numerous codons under purifying selection but no sites under significant positive selection. The RELAX test did not detect a significant change in the intensity of selection either in the concatenated dataset or in the *COIII* gene within the *P. bridgesii* clade.

## Discussion

### Mitogenome size and structure

The size of *Pomacea* mitogenomes ranges from 15,516 bp in *P. occulta* [77] to 16,373 bp in *P. diffusa* [78], placing *P. nobilis* (16279–16471) and *Pomacea* sp. (16355 bp) among the largest reported for the genus. However, the largest mitogenomes within Ampullariidae have been observed in *Pila*, particularly *P. virens* (16839 bp) and *P. globosa* (16903 bp) (Ayyagari, unpublished). The comparative mitogenome analyses also revealed that the proportion of intergenic regions in both *P. nobilis* and *Pomacea* sp. (∼4.8%) is higher than in other *Pomacea* species (2.43–3.46%), although lower than in *M. cornuarietis* (5.38%) and *Pila* (6.81–7.06%). Likewise, we detected differences in the location and size of their largest intergenic region (111 bp between *tRNA-Ala* and *tRNA-Arg*) when compared with other *Pomacea*, in which the largest intergenic region occurs between *COIII* and *tRNA-Lys* (47–50 bp in the *P. canaliculata* clade and 65–67 bp in the *P. bridgesii* clade) [21, 76, 79]. In *M. cornuarietis*, the largest intergenic region (145 bp) is located between *tRNA-Ala* and *tRNA-Arg* [74], whereas in *Pila* this region is longer (214–306 bp) and positioned between *tRNA-Gln* and *tRNA-Gly*. Thus, although length variation in PCGs, rRNAs, and tRNAs has been reported within Ampullariidae [21], differences in mitogenome size are primarily driven by the expansion of intergenic regions.

The mitogenomes of *P. nobilis* and *Pomacea* sp. include a putative 39 bp overlap between *ATP6* and *tRNA-Met*, a feature not previously reported in other Ampullariidae [21]. In contrast, the 7 bp overlap between *NAD4L* and *NAD4* identified in both species corresponds to a highly conserved feature across Ampullariidae [21, 76, 77, 79] and is similarly widespread in most Caenogastropoda [68, 80–82]. Other overlapping regions reported in *Pomacea*, such as *NAD1*/*tRNA-Pro* in *P. canaliculata*, *P. maculata* and *P. occulta* [76, 79], and *NAD5*/*tRNA-Phe* in *P. reevei*, *P. aulanieri* and *P. diffusa* [21], were not found in *P. nobilis* or *Pomacea* sp. Gene overlaps are relatively common in animal mitochondrial genomes and have been proposed to arise as a consequence of selective pressures toward genome minimization and compact gene organization [83–85]. Previous studies have also suggested that the transcriptional implications of overlapping regions may depend on whether the genes are encoded on the same or opposite strands, with opposite-strand overlaps potentially requiring less complex transcript processing [85]. Notably, the putative *ATP6/tRNA-Met* overlap identified in the *Effusa* representatives involves genes encoded on opposite strands. This may facilitate the evolutionary persistence of this overlap, which could represent a distinctive feature of the *Effusa* clade among available ampullariid mitogenomes.

### Mitochondrial base composition and protein-coding gene features

The overall base composition and skew patterns of *P. nobilis* and *Pomacea* sp. are consistent with other Ampullariidae, although both species exhibit the highest AT-skew and lowest GC-skew in the family. Slight differences in A + T content among *Pomacea* clades (*Effusa*, *P. bridgesii*, and *P. canaliculata*) reflect minor compositional variation, while differences among genomic features (CR> rRNAs> tRNAs> PCGs) are consistent with previously reported patterns in gastropods [80, 82, 86, 87] and may be related to functional differences [15].

We recovered consistent differences in the length of several PCGs within *Pomacea*, with both *Effusa* lineages analyzed here showing the smallest *ATP8* (156 bp), and an unusually long *ATP6* annotation (768 bp), together with the longest *NAD6* (498 bp), *COIII* (786 bp), and *NAD2* (1077 bp) recorded for the genus. Notably, *NAD1* and *NAD4* in both *Effusa* lineages match the lengths observed in the *P. bridgesii* clade, while *NAD5* aligns with that of the *P. canaliculata* clade. These patterns not only confirm the inter-clade differences reported between the *P. bridgesii* and *P. canaliculata* groups [21], but also indicate that the *Effusa* clade possesses a distinct pattern in PCG lengths, reinforcing its differentiation from the other two clades.

The annotation analyses suggested that the *ATP6* gene in both *P. nobilis* and *Pomacea* sp. may be unusually long (768 bp), exceeding typical lengths reported in both Ampullariidae and other Caenogastropoda, where values below 700 bp are most common (GenBank survey, Dec 2025). Read mapping showed high coverage (102–1376×) and no evidence of indels or ambiguous sites at the terminal region, supporting the integrity of the assembled region and continuity of the predicted open reading frame. Inspection of the nucleotide alignment showed no evidence of premature termination or frame disruption, revealing instead a variation in the stop codon positions across lineages, with a notable 3’ extension in the *Effusa* clade (Additional file 2). Gene boundaries were defined based on the longest uninterrupted ORF consistent with these read-mapping signals and conserved protein domains. InterPro analysis confirmed the presence of the conserved ATP synthase subunit a domain, and transmembrane prediction recovered the expected five helices, with additional residues forming a short C-terminal extension. Finally, alignment of ATP6 amino acid sequences across available *Pomacea* and other Ampullariidae revealed that all sampled representatives of the *Effusa* clade share this extension, whereas other species terminate earlier (Additional file 3).

One alternative explanation could involve the use of an incomplete stop codon, a feature rarely documented in Caenogastropoda [72, 80, 81, 88]. Under this scenario, *ATP6* could terminate closer to the adjacent *tRNA-Met* gene, and the apparent 39 bp overlap would reflect differences in gene boundary annotation rather than a true C-terminal extension. However, incomplete stop codons have not previously been reported in Ampullariidae, and the adjacent *tRNA-Met* is encoded on the opposite strand, potentially reducing constraints associated with the observed overlap [85]. Together, these observations are consistent with an extended ATP6 coding region in the sampled *Effusa* representatives, although this interpretation remains tentative and would benefit from future validation using transcriptomic data.

### Mitochondrial rRNAs, tRNAs, and control region features

The *12S rRNA* and *16S rRNA* of *P. nobilis* and *Pomacea* sp. are the largest reported within *Pomacea*, exceeding the ranges observed in the *P. canaliculata* and *P. bridgesii* clades, and their predicted secondary structures are consistent with previous reports in the genus [21] and other mollusks [41, 89, 90]. This includes the presence of specific features proposed for Caenogastropoda in the *16S rRNA* secondary structure, such as the stem-loop D13-D14 in Domain II, the development of Domain III and the presence of a bulge-stem-loop structure (G16) in Domain V [91], that are present in both *P. nobilis* and *Pomacea* sp. Although the comparison of the secondary structure of the mitochondrial RNA in *Pomacea* and other Caenogastropoda [41] revealed that they share the same number and arrangement of stems and loops, some differences were identified within *Pomacea*. Notably in 12S rRNA, loop 31 and stem 40 are larger in *P. nobilis* and *Pomacea* sp. than in *P. aulanieri* and *P. reevei* [21], suggesting that secondary-structure comparisons of the least conserved domains of 12S and 16S rRNA may be useful for distinguishing closely related species.

We found that in both tRNA for serine in *P. nobilis* and *Pomacea* sp., the clover-leaf secondary structure is modified by a large bulge replacing the typical stem-loop structure in the D-domain (Fig. 6). Among Ampullaridae, *tRNA-Ser1* exhibits this modified structure in *Pila*, *Marisa*, and most species of the *P. canaliculata* clade (*P. canaliculata*, *P. maculata* and *P. occulta*) [21, 76], but not in the *P. bridgesii* clade, where *tRNA-Ser2* is the modified tRNA [21]. The only other Ampullariidae with both tRNAs for serine modified is *P. reevei* of the *P. canaliculata* group [21]. Jühling et al. [92]. reported that the loss of the D-domain in *tRNA-Ser1* is widespread across many groups of animals, including Mollusca, whereas the modification of *tRNA-Ser2* is a rarer feature. Outside *Pomacea*, the loss of the D-domain in *tRNA-Ser2* has been reported in only a few Caenogastropoda [70, 80, 93, 94].

The resulting coverage plots (Fig. 7) show that reads map across the control region in all samples, but the coverage is heterogeneous, with evident peaks and drops, particularly within this region, and its length and structure should therefore be interpreted with caution. Despite these limitations, tandem repeat analysis suggested the presence of (TA)n motifs and short AT-rich repeats, with apparent differences among the three *P. nobilis* mitogenomes (Table 7). Similar AT-rich repeats have been described in the control region of other molluscan mitogenomes, such as in *Haliotis rubra* [95], where variation in repeat number has been proposed as a potential genetic marker. Additionally, differences in the number and size of predicted stem-loop structures were observed among *P. nobilis* and *Pomacea* sp. (Fig. 8), as well as in comparison with other species [21]. These observations suggest that the control region of *Pomacea* may harbor variable and potentially informative features, but further validation using long-read sequencing approaches will be necessary to confirm their biological relevance.

### Phylogenetic analysis and genetic distances

The phylogenetic analyses recovered Viviparoidea, Ampullarioidea, and Cyclophoroidea as monophyletic groups, consistent with previous mitogenomic studies [21, 70, 79, 80]. However, deeper caenogastropod relationships remain uncertain, and our discussion therefore focuses on relationships within Ampullariidae, where mitogenome data provide more robust resolution. The *Effusa* group was first recognized as a distinct clade within *Pomacea* based on molecular data from *P. guyanensis* and *P. glauca* [20]. Subsequent work identified *P. nobilis* as a third confirmed member of this clade [22], a species widely distributed across the Peruvian Amazon basin and characterized by its large and robust shell [22, 23]. The present study further expands the known diversity of the *Effusa* clade by identifying an additional lineage from the Aguaytía River, suggesting that species diversity within this group may be underestimated in the western Amazon.

Based on the whole mitogenomes, we found an intraspecific variation of 2.58% in *P. nobilis*, slightly lower than the variation reported by Ramírez et al. [23]. based on *COI* sequences from Loreto (3.17%). Similarly, the interspecific distance between *P. nobilis* and *Pomacea* sp. (7.23%) is comparable to the genetic distance between *P. maculata* and *P. occulta* (6.84%), also based on whole mitogenomes, supporting the genetic distinctiveness of the Aguaytía lineage relative to *P. nobilis*. Comparison of the *COI* sequence of *Pomacea* sp. from the Aguaytía River with publicly available *COI* sequences failed to reveal correspondence with any currently described species. Together with its phylogenetic placement within the *Effusa* clade, these results support the distinctiveness of the Aguaytía lineage. However, its precise taxonomic identity remains unresolved, and it is therefore treated here as an unidentified *Pomacea* lineage pending formal taxonomic assessment.

### Selection pressure analyses

The selection pressure analyses indicate that mitochondrial protein-coding genes in *Pomacea* are predominantly evolving under purifying selection, as reflected by *K*_*a*_*/K*_*s*_ ratios consistently below 1. This pattern is typical for animal mitochondrial genomes, where strong functional constraints maintain the integrity of oxidative phosphorylation genes [96]. Among the genes analyzed, *ATP8*, *NAD2*, and *NAD4L* showed comparatively higher *K*_*a*_*/K*_*s*_ ratios, suggesting relatively relaxed constraints and higher evolutionary rates. These results are consistent with previous observations that certain NADH dehydrogenase genes and ATP8 tend to evolve more rapidly in gastropod mitogenomes [97, 98]. The comparatively elevated rates in these genes support their potential utility as markers for population-level or phylogeographic studies in *Pomacea*. Although an episodic signal was detected in COIII within the *P. bridgesii* clade, this result was not significant after multiple testing correction and should therefore be interpreted cautiously.

## Conclusions

This study reports the first complete mitochondrial genomes of the *Effusa* clade, based on *P. nobilis* and a putative undescribed *Pomacea* lineage (*Pomacea* sp.). Comparisons with the *P. canaliculata* and *P. bridgesii* clades revealed notable differences in genome size, intergenic regions, gene lengths, tRNA secondary structures, whereas the control region showed potentially distinct features that require further validation. Similar associations between mitogenome organization and phylogenetic relationships have previously been reported for the *P. canaliculata* and *P. bridgesii* clades [21], and our results suggest that this pattern may extend to the *Effusa* clade. Together, these observations indicate that aspects of mitochondrial genome architecture may retain phylogenetic signal within *Pomacea* and reflect evolutionary modifications accumulated during the diversification of its major clades. Further exploration of additional lineages may reveal whether the mitogenomic features observed in *P. nobilis* and *Pomacea* sp. are widespread within the *Effusa* clade and could aid in refining our understanding of mitogenome evolution in *Pomacea*.

## Supplementary Information

Additional file 1: Closest BLASTn matches of the COI sequences obtained from *Pomacea *sp. specimens from the Aguaytía River against sequences available in GenBank. Additional file 2: Figure S1. Alignment of ATP6 nucleotide sequences across available *Pomacea *and other Ampullariidae, highlighting the variation in stop codon positions. Red boxes indicate the different termination sites: the ancestral stop codon position shared by *Pila*, *Marisa*, and most *Pomacea *species; the shifted stop codon in *P. diffusa*; and the distinct stop codon at the 3' extension terminal in the *Effusa *clade. Additional file 3: Figure S2. Alignment of ATP6 amino acid sequences across available *Pomacea *and other Ampullariidae. Additional file 4: Figure S3. Relative synonymous codon usage (RSCU) of the mitogenome of *Pomacea *sp.

## Data Availability

The COI and mitogenome sequences of *Pomacea nobilis* and *Pomacea *sp. are accessible at the NCBI GenBank (https://www.ncbi.nlm.nih.gov/genbank/; accession numbers PX672272-PX672275 and PX692142-PX692146, respectively). Raw Illumina reads are available in the NCBI Sequence Read Archive (SRA) under BioProject PRJNA1438792 (SAMN56548275–SAMN56548279).
